# SARS-CoV-2-related Guillain–Barré syndrome requires comprehensive diagnostic and therapeutic care

**DOI:** 10.25122/jml-2024-1002

**Published:** 2024-01

**Authors:** Ibrahim Abdelazim, Ainur Donayeva, Akzhunus Mannapova

**Affiliations:** 1 Department of Obstetrics and Gynecology, Faculty of Medicine, Ain Shams University, Cairo, Egypt; 2 Department of Normal Physiology, West Kazakhstan Marat Ospanov Medical University, Aktobe, Kazakhstan; 3 Department of Neurology, West Kazakhstan Marat Ospanov Medical University, Aktobe, Kazakhstan

## DEAR EDITOR

We appreciate the opportunity to address the comments and questions raised regarding our case report on Guillain–Barré syndrome (GBS) and Miller Fisher syndrome (MFS) following SARS-CoV-2 infection [1]. Our case report focused on presenting the relationship between GBS and COVID-19, aiming to highlight the potential neurological complications associated with SARS-CoV-2 infection.

### The case of Patient 1

Patient 1 presented with a history of urinary tract infection (UTI) 3 weeks before the onset of GBS symptoms caused by *Escherichia coli* (*E. coli*), which accounts for 80% of uncomplicated UTIs [2]. While *E. coli* UTIs are not traditionally associated with GBS, we conducted extensive testing to rule out more common pathogens linked to GBS, such as *Campylobacter jejuni, Mycoplasma pneumoniae*, and various viruses [3], before establishing a diagnosis of SARS-CoV-2-related GBS. The absence of cerebrospinal fluid (CSF) testing, as per the Brighton criteria, was a limitation of this study. We recognize the critical role of CSF testing in diagnosing GBS; however, a systematic review highlighted that a significant percentage of adults (26.6%) did not undergo this test, reflecting a wider issue in the current clinical scenario [4]. This is a relevant limitation because CSF testing is very important for accurate diagnosis and subsequent treatment, especially in critically ill patients.

### The case of Patient 2

The diagnosis of MFS in Patient 2 was based on neurological examination, which showed bilateral facial paresis and ptosis, with dysphonia, dysphagia, and quadriplegia (Medical Research Council scale [5], 1/5 proximal and 0/5 distal), hypotonia, absent hand and foot reflexes with a positive Babinski sign. The electroneurography showed acute demyelinating polyneuropathy (nerve latency delay affecting sensory and motor nerves, decreased conduction velocity, and amplitude of the action potential). The CSF examination following lumbar puncture showed elevated CSF protein without pleocytosis [6] and increased immunoglobulin (mainly anti-GQ1b IgG)/albumin ratio [7]. The case of Patient 2 may be considered an atypical form of MFS based on bilateral ptosis, decreased tendon reflexes, and elevated CSF protein levels (increased CSF immunoglobulin (mainly anti-GQ1b IgG) [7] without pleocytosis [6]). The diagnosis of an atypical MFS in Patient 2 was confirmed by the increased CSF immunoglobulin (anti-GQ1b IgG), which has 85% sensitivity and 100% specificity for diagnosing MFS [8]. Atypical forms of MFS have also been reported with unilateral or bilateral ophthalmoplegia, bilateral abducens palsy, and/or isolated bilateral ptosis [9,10]. Patient 2 was mechanically ventilated when she developed severe respiratory distress, and she died because of respiratory failure following severe bilateral pneumonia and interstitial pneumonitis caused by SARS-CoV-2, despite all supportive treatments before any attempts for extracorporeal membrane oxygenation and/or plasma exchange [1].

### The immune response to SARS-CoV-2

The immune response to SARS-CoV-2 involves the production of antibodies against the lipo-oligosaccharides of the virus, which are similar in structure to gangliosides (GQ1b, GM1, and GD1a) located on the neuronal cell surface [5]. The antibodies of the host against the lipo-oligosaccharides of the SARS-CoV-2 membrane cross-react with the gangliosides (GQ1b, GM1, and GD1a) located on the neuronal cell surface, with the subsequent autoimmune destruction of myelin sheaths and/or nerve axons [3]. Antibodies against GM1 or GD1b were detected in classic GBS, whereas antibodies against GQ1b were detected in MFS [5].

## CONCLUSION

Our case report aimed to inform healthcare providers about the potential neurological implications of SARS-CoV-2 infection. However, it is important to acknowledge the limitations of our findings. The diagnosis of GBS in Patient 1 was based on clinical presentation and nerve conduction studies without the key confirmation of elevated CSF protein, as outlined by the Brighton criteria. In addition, Patient 2 may be considered an atypical form of MFS rather than classical MFS. Finally, we hope that our reply highlighted the relationship between GBS and SARS-CoV-2 for healthcare providers, emphasizing the need to be prepared for patients with GBS and a rapidly progressive course requiring immediate, aggressive, and comprehensive treatment. We hope this response clarifies the methodology and considerations behind our case report. It underscores the importance of continued research and discussion within the scientific community to deepen our understanding of the neurological implications of COVID-19.
